# Neural mechanisms and social support for mental health in old age by martial arts exercise

**DOI:** 10.3389/fpsyg.2025.1733310

**Published:** 2026-01-09

**Authors:** Yong Jiang, Pengsong Li, Yulong Yang, Liqing Liu, Haibin Liu, Yan Zhu, Fengshan Gao, Yanze Li, Suheng Li, Junjie Wang, Qingyong Wang, Shuye Yang

**Affiliations:** School of Kinesiology and Health Promotion, Dalian University of Technology, Dalian, China

**Keywords:** cognitive function, martial arts, mental health, neural mechanisms, older adults, social support

## Abstract

The increasing global aging population presents significant challenges related to cognitive decline, mental health disorders, and social isolation. Martial arts exercise emerges as a multifaceted intervention promoting mental health and cognitive vitality among older adults by integrating physical activity, cognitive engagement, and social interaction. This review synthesizes evidence on the neural mechanisms underlying the benefits of martial arts, highlighting their role in enhancing brain-derived neurotrophic factor (BDNF) expression, neuroplasticity, and neural connectivity, which support improved executive functions, memory, and emotional regulation. Both hard martial arts and soft practices, such as Tai Chi, offer distinct advantages in addressing age-related cognitive and psychosocial challenges. Additionally, martial arts foster strong social support systems, reducing loneliness and enhancing emotional resilience through community engagement and shared achievement. Physical and functional benefits, including improved strength, balance, and cardiovascular health, further contribute to overall well-being. Despite promising results, current studies are limited by heterogeneity in martial arts styles, short intervention durations, and variable methodologies. Future research should focus on long-term, standardized interventions employing advanced neuroimaging and biomarker assessments to better elucidate mechanisms and optimize training protocols. Integrating martial arts into health promotion strategies holds substantial potential for enhancing mental health, cognitive resilience, and quality of life in aging populations.

## 1 Introduction

The global aging population presents significant challenges to public health, as older adults often face cognitive decline, increased risks of anxiety and depression, and social isolation, negatively impacting their quality of life and independence (Valdés-Badilla et al., 2021). Therefore, identifying effective interventions to promote mental health and cognitive resilience is crucial. While physical exercise is widely recognized for mitigating cognitive decline and enhancing psychological well-being, recent research suggests that martial arts offer a promising, multifaceted approach by combining physical activity with cognitive stimulation and social engagement (Guo et al., 2016; Sullivan et al., 2024a; Sullivan et al., 2024b). However, the neural, psychosocial, and embodied mechanisms through which this works are not sufficiently consolidated. Accordingly, this review critically considers evidence on the effects of martial arts on neuroplasticity, cognitive functioning, and psychosocial adaptation in old age from the perspectives of neuroscience, embodied cognition technologies, and gerontology.

Martial arts, including Olympic sports like boxing, judo, karate, and traditional practices like Tai Chi, combine aerobic exercise, complex motor skills, cognitive challenges, controlled breathing, and social interaction (Gomboev et al., 2023). This comprehensive approach targets physical fitness, brain health, emotional regulation, and social connections, addressing the multifaceted nature of healthy aging. From a neurobiological perspective, martial arts training affects mental health by modulating brain-derived neurotrophic factor (BDNF), essential for synaptic plasticity and neurogenesis (Hola et al., 2024). BDNF levels decline with age, contributing to memory and cognitive decline. However, martial arts participation has been shown to increase BDNF levels, promoting neuroplasticity. Studies on older adults engaging in Judo and Tai Chi have demonstrated higher BDNF levels, improved cognitive performance, and structural brain changes, including increased gray matter in the hippocampus and prefrontal cortex (Bu et al., 2010; Sungkarat et al., 2018).

Martial arts involve complex motor movements and cognitive functions, requiring practitioners to memorize sequences, anticipate actions, make rapid decisions, and sustain attention (Kotarska et al., 2019). These motor-cognitive demands engage neural circuits related to executive control, working memory, and attention, which decline with age. Neuroimaging studies show that regular practice increases cortical thickness and functional connectivity between regions responsible for cognitive flexibility and self-awareness (Lazar et al., 2005). These changes enhance multitasking, processing speed, and overall cognitive performance, essential for maintaining independence and quality of life in older adults (Liu et al., 2021). Motor sequencing, attentional switching, inhibition, and visuospatial processing were all more challenging in martial arts relative to traditional exercise activities such as walking or resistance training. Various studies have demonstrated that these types of cognitively challenging forms of exercise induce better enhancements in executive function and working memory than low-cognitive-load activities.

Beyond neurological benefits, martial arts improve psychological and social well-being. Hard martial arts, such as Karate, Taekwondo, and Judo, have been linked to reduced anxiety and depression, enhanced self-esteem, and greater life satisfaction (Valdés-Badilla et al., 2022a; Gastoł et al., 2024). These results can be biased by confounding factors, including baseline physical conditions in the study population. They may differ among culturally or ethnically diverse older adults engaged in different forms of martial arts. The communal environment of martial arts classes fosters social connectedness, alleviating loneliness in aging populations. Gender and regional differences in mental health outcomes are also a factor, since women and older adults from East Asian regions display different engagement patterns, motivational factors, or psychological responses to practicing martial arts than a Western cohort.

Group training provides meaningful social interactions, mutual encouragement, and shared accomplishments, which strengthen emotional resilience and motivation for continued physical activity (Wang et al., 2023). The structured progression in martial arts, such as belt advancement, boosts confidence and psychological well-being (Sullivan et al., 2024a; Sullivan et al., 2024b). Nonetheless, differences in neuroimaging procedures (fMRI pre-processing and ROI selection), together with the known issue of variability from serum BDNF assays, prevent direct comparison between studies. Current neurobiological data should therefore be interpreted carefully.

Based on embodied cognition theory, which posits that cognitive functions are firmly rooted in sensorimotor experience, martial arts offer a rich combination of movement flow, attention management, emotional, and intersubjective interactions. Such embodied practices drive neuroplastic change within motor, cognitive, and socio-emotional circuits, thereby providing a multilayered trajectory for successful aging. The virtues of martial arts can be examined through the framework of embodied cognition and neuroplastic adaptation. These theories imply an interdependence of motor and cognitive systems and that the neural architecture and emotional self-regulation are influenced by sensorimotor experiences. Using these systems together helps to understand why martial arts produce multidimensional benefits in mental health, as it incorporates elements of the cognitive, motor, and emotional types together. This review examines the neurobiological, psychological, social, and physical benefits of martial arts for aging individuals. We discuss training recommendations, literature limitations, and suggest future research directions to advance this field. Integrating insights from neuroscience, psychology, and gerontology, martial arts emerge as a holistic approach to healthy aging, offering significant potential to enhance mental health and quality of life for the aging global population.

## 2 Review methodology

This is a narrative review that attempts to consolidate existing knowledge across the neural, cognitive, and social system dimensions of martial arts as these relate to mental health in older adults. A systematic search was carried out in the databases of PubMed, Scopus, Web of Science, and Google Scholar by using the terms: martial arts; Tai Chi; older adults; mental health; cognition; neuroplasticity; social support; BDNF. The search included studies from 2000 to 2024. Inclusion criteria: (1) peer-reviewed articles, (2) 55 years or older, (3) martial arts or Tai Chi as the intervention, and (4) mental health, cognition, neural mechanisms, and psychosocial well-being outcomes. Exclusion criteria: (1) non-peer-reviewed; (2) without mental or neural results; and (3) not related to martial arts interventions. Manual reference list screening was performed to supplement articles.

## 3 Neural mechanisms

### 3.1 Older adults

**Figure 1:**
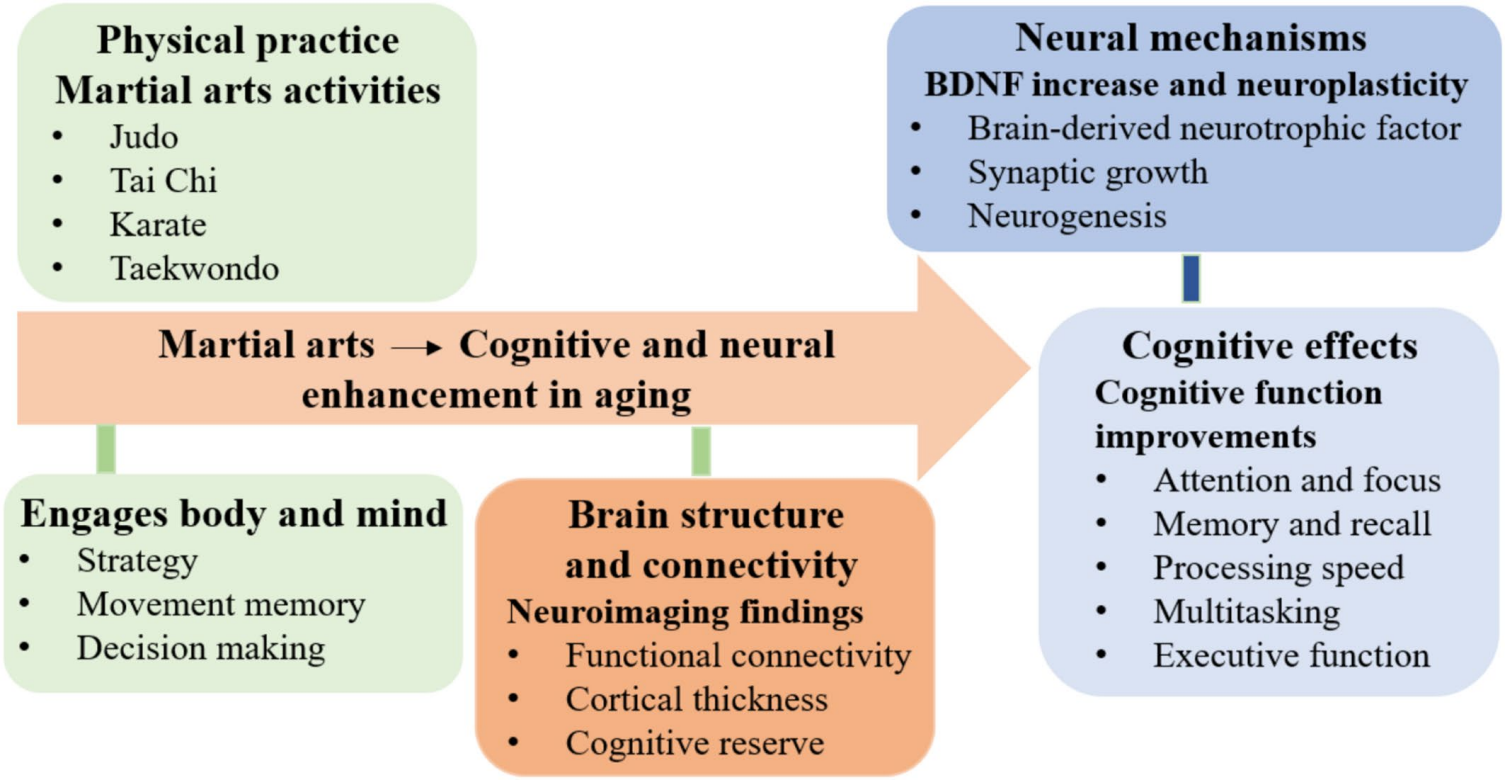
Neurocognitive benefits of martial arts in aging: enhanced BDNF, improved executive functions, and strengthened brain connectivity.

Martial arts training has gained recognition for its positive effects on brain function in older adults, primarily by engaging multiple neural mechanisms (Ciaccioni et al., 2024) (Figure 1). The complex nature of martial arts, requiring strategy, anticipation, decision-making, and coordinated physical movements, stimulates various cognitive domains. This comprehensive cognitive-motor engagement supports improvements in attention, memory, and executive functions, which often decline with age. Martial arts also promote neural plasticity by enhancing BDNF levels, facilitating synaptic growth, and neuronal survival (Campanella et al., 2024). These neurobiological changes contribute to maintaining and even improving cognitive performance, making martial arts a promising intervention to counteract age-related neural decline and promote healthy brain aging (Table 1).

**Table 1:** Neural mechanisms underlying the promotion of mental health in older adults through martial arts exercise.

Neural mechanism	Observed mental health benefit	Social/behavioral effects	Martial Art modality	Reference
Increased hippocampal volume	Improved memory and reduced risk of dementia	Increased social engagement and group cohesion; improved balance and confidence	Tai Chi, Karate, Aikido	Bu et al. (2010)
Enhanced functional connectivity in DMN	Better cognitive flexibility and self-awareness	–	Tai Chi	Lin and Zhao (2025)
Upregulation of BDNF expression	Mood enhancement, neurogenesis, antidepressant-like effects	Emphasis on cooperation and mutual respect	Tai Chi, Kung Fu	Maillane-Vanegas et al. (2020)
Reduced amygdala reactivity	Lower anxiety and improved emotional regulation	Promotes social bonding during group practice	Tai Chi, Qigong	Guo et al. (2016)
Cortical thickness maintenance	Delay in age-related cortical atrophy	Strong group identity and motivation	Aikido, Karate	Bhattacharya et al. (2022)
Decreased HPA axis activity	Reduced cortisol, stress response attenuation	Promotes social bonding during group practice	Tai Chi, Qigong	Yeung et al. (2018)
Increased prefrontal cortex activation	Improved executive functioning and decision-making	Emphasis on cooperation and mutual respect	Tai Chi, Judo	Chen et al. (2022)
Enhanced sensorimotor integration	Better balance, coordination, and fall prevention	Emphasis on cooperation and mutual respect	Tai Chi, Judo	Li et al. (2024)
Improved white matter integrity	Enhanced cognitive processing speed	–	Tai Chi	Shao et al. (2024)
Greater dopamine release	Mood elevation and reward pathway stimulation	Structured progression fosters goal-setting and social recognition	Taekwondo, Karate	Humińska-Lisowska (2024)

### 3.2 BDNF increase and neuroplasticity

Tai Chi Chuan, a meditative martial art with slow, flowing movements, has gained attention for its neuroprotective effects in older adults. It elevates brain-derived neurotrophic factor (BDNF), a protein crucial for neuronal survival, synaptic plasticity, and cognitive function (Lin et al., 2025). BDNF supports neuroplasticity, which declines with age. Both acute and long-term Tai Chi practice have been shown to increase peripheral BDNF levels, enhancing cognitive performance and brain health (Tao et al., 2017). By promoting neuroplasticity, Tai Chi helps preserve cognitive abilities and may slow age-related decline, offering an accessible intervention for brain health and mental well-being in aging populations.

### 3.3 Cognitive improvement through complex motor and cognitive engagement

Martial arts training combines complex motor skills with demanding cognitive processes, offering a dual challenge that benefits cognitive health in older adults (Pujari, 2024). Practitioners must learn intricate movement sequences, maintain attention, anticipate actions, and make rapid decisions. This integration of motor coordination and cognitive functions stimulates brain regions related to executive control, working memory, and attentional regulation. Unlike conventional exercise, martial arts engage multiple cognitive domains, fostering neuroplasticity and enhancing mental agility, which are vital for daily functioning (Pujari, 2024). These mental challenges are associated with structural brain changes, including increased cortical thickness and volume in regions responsible for executive function (Cui et al., 2021).

### 3.4 Enhancement of executive functions

Martial arts training significantly enhances executive functions, including planning, attention, working memory, problem-solving, and inhibitory control. It requires integrating complex motor skills with strategic thinking, quick decision-making, and emotional regulation, which activate the prefrontal cortex and improve mental flexibility and self-control (Hu et al., 2024). In addition to boosting physical fitness, regular martial arts practice sharpens cognitive abilities essential for managing daily tasks and overcoming challenges. Through structured routines, practitioners develop focused attention, impulse control, and perseverance, skills that improve cognitive control in various life areas. Research shows that martial arts benefit individuals of all ages, enhancing executive function and promoting brain health (Harwood-Gross et al., 2021).

### 3.5 Neural connectivity and brain network modulation

This low-impact exercise combines slow movements, focused attention, and deep breathing to enhance communication between different brain regions (Cui et al., 2021). Neuroimaging studies show that regular Tai Chi practice strengthens connectivity within networks like the default mode network, sensorimotor network, and executive control network, promoting better coordination, balance, and cognitive function, while supporting brain plasticity and functional integration (Cerna et al., 2024).

Additionally, Tai Chi reduces neural noise and enhances synchronization across brain networks, improving emotional regulation and stress resilience. Its mindful, meditative aspects foster neuroplasticity, benefiting cognitive processing speed and memory (Wang et al., 2023). These brain network modulations support healthy aging and may protect against cognitive decline, positioning Tai Chi as a promising intervention for maintaining brain health throughout life.

### 3.6 Controversies and contradictions in current evidence

Current evidence of martial arts benefits is contemporaneous with some controversy and contradictions. While many studies report elevated cognitive and emotional outcomes resulting from participation in martial arts training, other studies have conflicting definitions and results (Kujach et al., 2022; Oh et al., 2025). Short-term results of less than 12 weeks of intervention will report minimal or no improvement in executive function compared to aerobic exercise (Wang et al., 2025). The issue of what causes the enhanced brain responses that have been seen is still up for debate. It is mostly caused by the cardiovascular nature of the exercise, though one could argue that martial arts practice typically involves mental engagement that is both cognitive and meditative (Pujari, 2024). These contradictions can best be assessed and resolved by studies that attempt to compare the different styles of martial arts practice, different exercise intensities, and different degrees of cognitive involvement.

## 4 Social support and psychological wellbeing

Martial arts cultivate strong social support networks, which are crucial for enhancing mental and emotional well-being. From a social neuroscience point of view, the underpinnings of social connection are allegedly associated with oxytocinergic modulation, activation in reward circuitry (ventral striatum, mPFC), and decreased amygdala reactivity. These mechanisms provide biobehavioral pathways by which social support promotes stress regulation and psychological resilience.

Training in group settings allows individuals to motivate one another, build trust, and work toward shared goals, creating a supportive environment that alleviates loneliness and stress, particularly among older adults or those facing social isolation (Oh et al., 2025). Partner exercises, group drills, and collective challenges strengthen social bonds and foster a sense of belonging. Social support can be categorized as structural support (size, density, and frequency of social networks) and functional support (perceived quality, emotional closeness, and instrumental help). These dimensions have different psychological and neurobiological implications and ought not to be treated as interchangeable. Yet supportive social contexts have been interpreted as exerting buffering effects on neurobiological responses to stress in several lines of evidence from neuroimaging studies, lending credibility to the biological plausibility of social support as a protective factor against poor mental health.

### 4.1 Socialization and community engagement

Martial arts provide older adults with valuable opportunities for socialization and community engagement. Group classes offer a structured environment for regular connection, friendship, and shared experiences, combating loneliness and isolation, which are common in aging (Miller et al., 2022). This social interaction fosters a strong sense of belonging and enhances teamwork and communication, strengthening social bonds.

Participation in martial arts also connects older adults to broader community networks through events, demonstrations, and activities (Sullivan et al., 2024a; Sullivan et al., 2024b). These opportunities promote intergenerational interaction and cultural exchange, enriching the social fabric and enhancing quality of life. By combining physical activity and social involvement, martial arts offer a comprehensive approach to healthy aging, supporting both mental wellness and ongoing community participation.

### 4.2 Enhancement of self-worth and psychological well-being

Martial arts training enhances self-worth by fostering achievement, discipline, and personal growth. As practitioners progress and overcome challenges, they build confidence and a stronger self-identity. The structured nature of martial arts encourages goal setting and perseverance, boosting self-esteem and improving resilience in facing life’s stresses. Beyond improving self-esteem, martial arts promote psychological well-being by providing stress relief and emotional regulation. The mindful focus on movement, breathing, and mental discipline cultivates a calm, centered mindset (Oh et al., 2025). Regular practice can alleviate anxiety and depression, offering a constructive way to manage emotions. These psychological benefits make martial arts a holistic practice that nurtures both body and mind, supporting mental health and overall life satisfaction (Ciaccioni et al., 2024).

## 5 Mental health benefits

Martial arts provide significant mental health benefits by combining physical activity with mindfulness and discipline. Regular training reduces stress, anxiety, and depression by promoting endorphin release and encouraging focused breathing and movement. The structured routines and goal-oriented progression foster a sense of purpose and achievement, improving mood and emotional stability. Martial arts also enhance concentration, mental clarity, and cognitive resilience (Moore et al., 2020). To sort the level of evidence, we separated RCTs from longitudinal and cross-sectional studies due to their varying methodological rigor and capacity for causal inference.

In addition to individual benefits, martial arts create a supportive community environment that promotes social connection and belonging, essential for mental well-being. The focus on respect, self-control, and emotional regulation equips practitioners with coping skills for managing everyday challenges. These combined physical, cognitive, and social benefits make martial arts an integrated approach to improving and maintaining mental health across all age groups (Table 2).

**Table 2:** Martial arts benefits for health.

Mental health benefit	Description	References
Anxiety reduction	Martial arts (Karate, Tai Chi) reduce anxiety by combining physical activity, mindfulness, and stress regulation	Mahalakshmi and Shaji (2024)
Stress management and Emotional regulation	Martial arts use mindfulness, breathing, and meditative movements (Tai Chi, Kung Fu) to improve emotional regulation and reduce stress; hard styles help release pent-up emotions	Martín-Rodríguez et al. (2024)
Cognitive function enhancement	Martial arts enhance motor and cognitive skills, improving memory, attention, and executive function, especially in older adults	Dunsky (2019)
Quality of life and well-being	Martial arts improve quality of life by boosting fitness, mental focus, and social support, enhancing self-worth and reducing loneliness in older adults	Abbott and Lavretsky (2013)
Social support and connectedness	Group martial arts foster social interaction, reducing isolation and loneliness in older adults, while camaraderie boosts emotional resilience and motivation	Chan et al. (2017)
Improved mood and depression reduction	Regular martial arts practice reduces depression and improves mood by fostering mastery, achievement, and social belonging; Tai Chi and Ba Duan Jin are especially effective for older adults	Hola et al. (2024)

### 5.1 Anxiety and stress reduction

Martial arts training effectively reduces anxiety and stress by combining physical activity with mindfulness and controlled breathing. The rhythmic movements and focused attention activate the parasympathetic nervous system, calming the body and mind, which leads to a lower heart rate and reduced cortisol levels (Renaghan et al., 2023). Additionally, martial arts provide a healthy outlet for releasing tension and emotions, helping practitioners feel more relaxed and centered.

Beyond physiological benefits, martial arts promote mental discipline and emotional regulation, equipping individuals to manage stress more effectively. The structured environment and goal-setting foster a sense of control and achievement, counteracting anxiety. Regular participation also builds resilience, self-confidence, and a positive mindset, supporting long-term stress management and emotional well-being. Specifically, Karate training has been shown to reduce depression and improve emotional well-being in older adults, with participants experiencing significant improvements compared to control groups (Moore et al., 2020). These findings suggest that martial arts’ integrated physical, cognitive, and emotional demands contribute to better stress management and anxiety reduction.

### 5.2 Improved wellbeing

Martial arts significantly enhance overall well-being by integrating physical, mental, and social elements. Regular training improves physical fitness, coordination, and balance, boosting energy and reducing chronic disease risks (Sun et al., 2024). The mental focus and mindfulness foster emotional stability and mental clarity, promoting a balanced state of mind. Additionally, martial arts provide a strong sense of community and social engagement, positively impacting emotional health. The support from instructors and peers enhances motivation, belonging, and self-esteem. These combined physical, psychological, and social benefits elevate quality of life, making martial arts a powerful practice for improving well-being at any age (Oh et al., 2025).

### 5.3 Emotional state enhancement

Karate practice significantly enhances emotional well-being by cultivating self-discipline, focus, and emotional control. The rigorous training and repetition involved help practitioners channel emotions like anger and frustration into productive energy, fostering calmness and mental clarity. Through consistent practice, Karate develops patience and resilience, enabling individuals to better regulate their emotions and respond thoughtfully to stressful or challenging situations (Potoczny et al., 2021).

**Figure 2:**
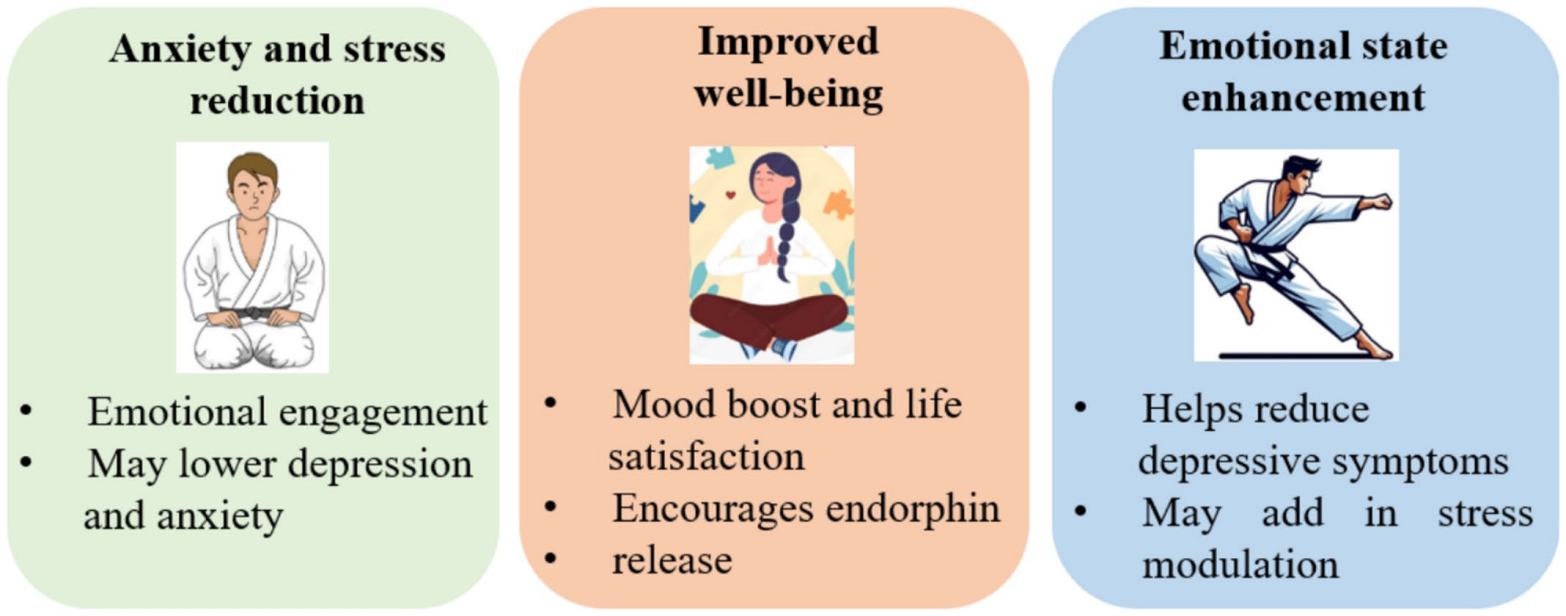
Mental health benefits of martial arts training in older adults.

Moreover, Karate’s structured environment and the strong sense of community support positive emotional growth. Achieving new belts and mastering techniques instils a sense of accomplishment and boosts self-confidence, which uplifts overall mood. The combination of physical exertion, mental focus, and social interaction in Karate contributes to improved emotional balance and psychological resilience (Figure 2).

## 6 Physical-functional health

Olympic combat sports (OCS) such as boxing, judo, karate, taekwondo, fencing, and wrestling offer extensive physical-functional health benefits by combining cardiovascular endurance, strength, flexibility, and coordination. These sports improve motor skills, cardiovascular health, bone density, and muscular endurance, reducing chronic disease risks and promoting long-term mobility and independence (Muñoz-Vásquez et al., 2023). OCS also enhances neuromuscular function, reaction time, and body awareness through complex, dynamic movements, which are crucial for injury prevention and daily activities. These physical and functional benefits foster a resilient body, supporting both athletic performance and overall health across age groups.

### 6.1 Muscle strength and cardiorespiratory capacity

OCS improve muscle strength by engaging participants in high-intensity, resistance-based movements that target multiple muscle groups. Sports like boxing, judo, karate, taekwondo, fencing, and wrestling involve dynamic actions such as striking, grappling, and throwing, which enhance both upper and lower body power (Hernandez-Martinez et al., 2025). These strength gains improve functional performance, aid injury prevention, and support overall physical health. In addition to muscle strength, OCS training enhances cardiorespiratory capacity through sustained aerobic and anaerobic exertion. The combination of rapid bursts and continuous movement improves heart and lung function, boosting endurance and stamina. Enhanced cardiorespiratory fitness promotes better oxygen delivery to muscles and organs, supporting recovery and vitality for athletes and the general population (Cheng et al., 2019).

### 6.2 Balance, agility, and flexibility

Balance and agility are crucial for fall prevention, especially among older adults. OCS such as boxing, judo, karate, taekwondo, fencing, and wrestling enhance postural control and coordination through dynamic movements. Training drills that involve rapid direction changes, weight shifts, and precise footwork improve neuromuscular responses and reflexes, reducing fall risk and enhancing stability (Valdés-Badilla et al., 2022b). Additionally, OCS improves flexibility, supporting joint mobility and muscle elasticity, which helps maintain independence and reduce age-related stiffness. These benefits promote safer movement and a better quality of life across all ages.

### 6.3 Training recommendations and adherence

OCS programs for older adults focus on gradual progression, safety, and adaptation to individual fitness levels. Training incorporates low-impact techniques, balance exercises, and controlled movements to minimize injury risk and enhance functional benefits. Instructors customize routines to address age-related challenges like joint stiffness and reduced cardiovascular capacity, ensuring comfortable participation. Warm-ups, cool-downs, flexibility, and strength training further support health and injury prevention (Cabo et al., 2025). Group classes with clear goals, regular feedback, and positive reinforcement promote sustained engagement. Emphasizing mental and social benefits alongside physical improvements helps maintain motivation, making OCS an effective and enjoyable approach to healthy aging and improved quality of life.

## 7 Bridging neural, social, and psychological pathways

**Figure 3:**
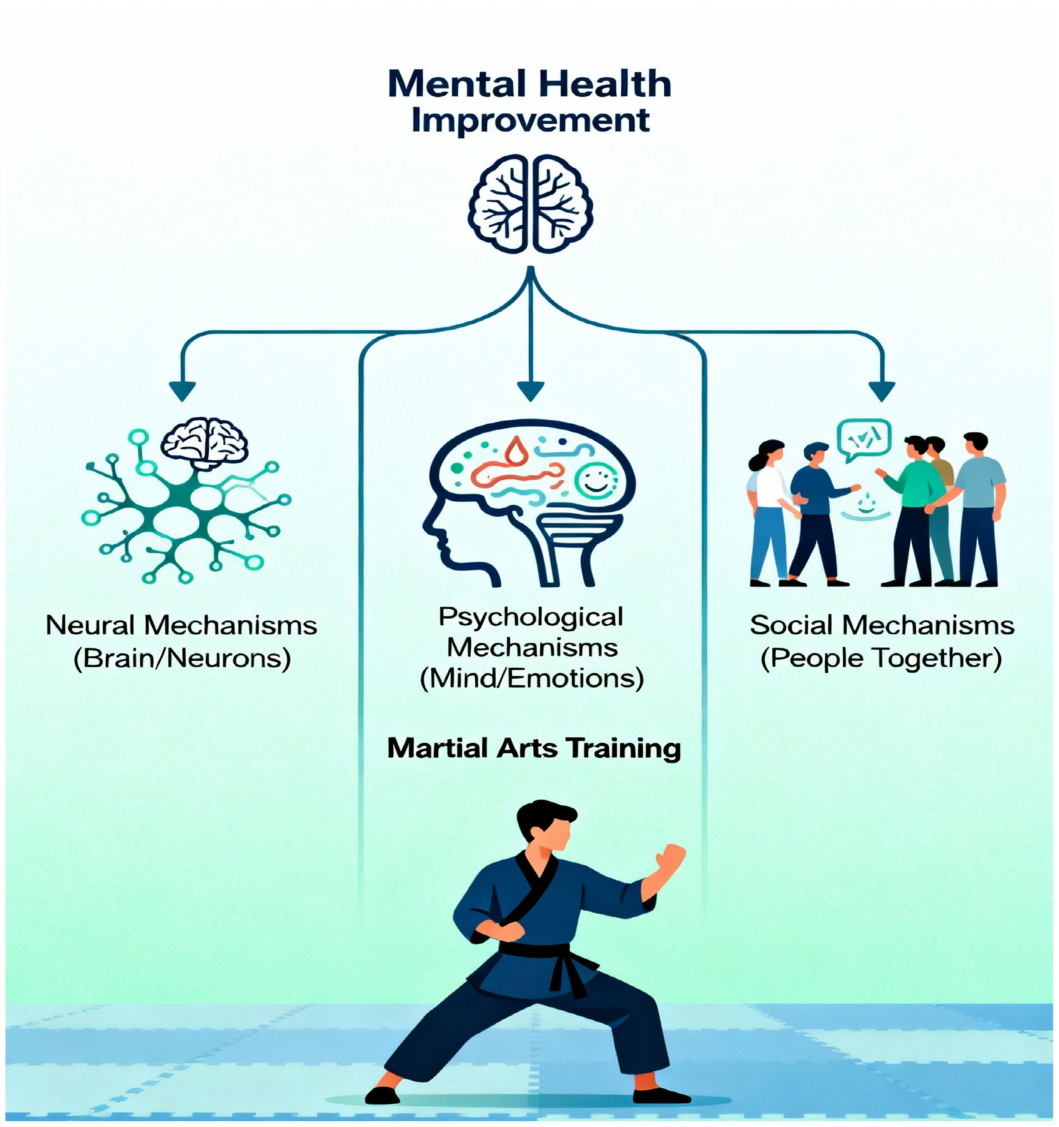
Neural, psychological, and social mechanisms of martial arts benefits in older adults.

The influence of martial arts on the mental health of older adults is most appropriately understood in an integrated biopsychosocial framework that links neural, psychological, and social mechanisms (Willhite and Becht-buss, 2025) (Figure 3). Rather than evoking their effects through isolated physiological changes, martial arts interventions incorporate diverse, interacting pathways that collectively promote cognitive resilience to adversity, emotional stability, and social health. At the neural level, regular practice of martial arts enhances neuroplasticity, cortical connectivity, and brain-derived neurotrophic factor (BDNF) expression, all of which are important for memory consolidation, learning, and emotional regulation (Hola et al., 2024).

These neurobiological adaptations form a basis for improved executive function, attention, and self-regulation. This improved connectivity in prefrontal and hippocampal pathways promotes the neural substrates of decision making and emotional control, which are so frequently rendered deficient late. Psychologically, these neurological changes come to manifest themselves in terms of improved self-efficacy, improved mood control, and cognitive flexibility (Sridhar et al., 2023). The mastery-oriented structure of martial arts, progressive acquisitions of skills, goal setting, and feedback nurture intrinsic motivation and self-worth. The sense of achievement and self-discipline derived from regular training serves to ameliorate symptoms of anxiety and depression, thus promoting emotional resilience and mental balance (Ambroży et al., 2024). This psychological empowerment, in turn reinforces neural adaptation as neural potentiation comes to accompany sustained practice, which leads to less neural inhibition through stress. Socially, martial arts are beneficial in fostering institutional connectedness and mutual support, factors that insulate against loneliness and isolation, among the most potent risk factors for cognitive and emotional decline in the aged (Harwood-Gross et al., 2021).

Group training engenders empathy, cooperation, and social identity, and recruits the reward mechanism and oxytocin-mediated pathways associated with positive affect and stress release. The interaction of neural health and social connectedness congeals into a synergistic feedback loop in which social connectedness enhances group neural activity and vice versa (Kim and Sul, 2023). Collectively, all these interdependent factors imply that martial arts serve not merely as a form of exercise but as a multidimensional neuro psychosocial intervention. By simultaneously acting at the level of the brain, the mind, and the social environment, martial arts promote holistic well-being and could serve as an inexpensive and easily accessible method of mitigating cognitive and emotional deterioration in ageing.

Previous work has shown that formal physical activity interventions can have a beneficial impact on psychological and physiological parameters, including mood, emotional resilience, and neurochemical balance (Kanani et al., 2025). The integration of these findings within the current context is important in connecting psychosocial engagement through sports video game play to the wider theoretical framework associated with exercise and mental health benefits. This association provides evidence that engagement in the physical domain, whether through traditional exercise or more novel means such as sports video games, may have positive indirect effects on overall psychological well-being.

## 8 Types of martial arts

The 2024 Paris Olympics will feature six core OCS, each with its unique characteristics and rich history. Boxing, one of the oldest Olympic combat sports, has been part of the modern Olympic program since 1904, focusing on precise punches, footwork, and defensive maneuvers, emphasizing speed, power, and tactical skill (Walsh, 2008).

**Figure 4:**
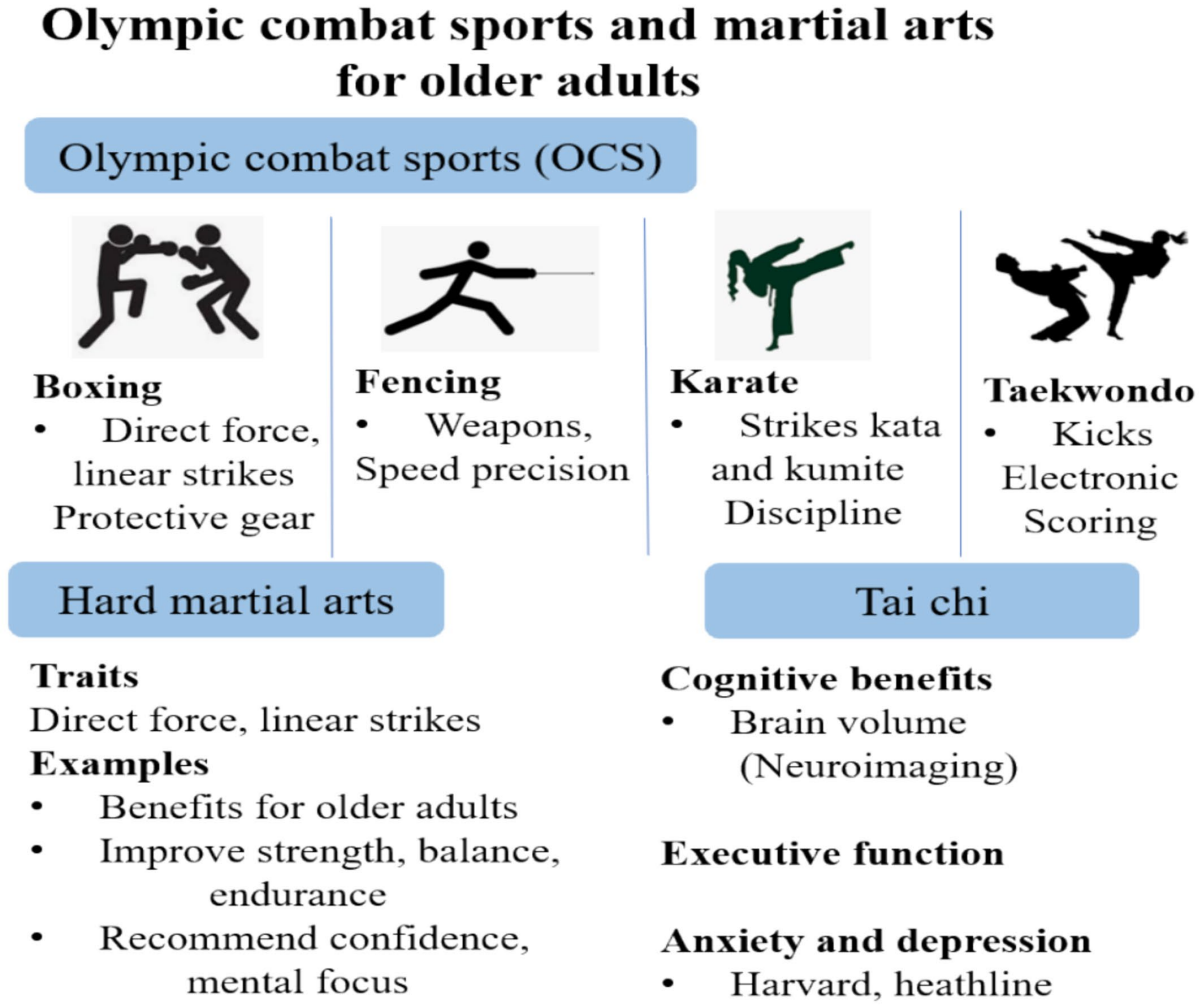
Visual overview illustrates the six Olympic combat sports and hard martial arts and Tai Chi, highlighting their physical, cognitive, and emotional benefits, especially for aging populations.

Judo, introduced in 1964, combines throwing and grappling techniques, using leverage and balance to subdue opponents. Karate made its Olympic debut in Tokyo 2020, blending physical agility, striking, and blocking. Taekwondo, an Olympic sport since 2000, is known for dynamic kicking techniques and fast-paced combat (Tokarski, 2012). Wrestling, one of the original Olympic sports, includes Greco-Roman and freestyle styles, both requiring exceptional strength, endurance, agility, and strategic thinking. These Olympic combat sports highlight a blend of tradition, athleticism, and mental strategy, making them key attractions of the Paris 2024 Games (Latyshev et al., 2020) (Figure 4).

### 8.1 Hard martial arts

Hard martial arts focus on direct, forceful techniques designed to overpower or incapacitate an opponent through strength, power, and speed (Lahti et al., 2016). These styles emphasize linear, explosive movements like punches, kicks, and strikes delivered with maximum impact. The philosophy behind hard martial arts is generating and applying external force to dominate in combat. Training involves conditioning the body to withstand and deliver powerful blows, developing strength, speed, and precision.

Techniques in hard martial arts are executed with rigid stances and firm strikes aimed at vulnerable body targets. Extensive training in offensive and defensive maneuvers builds muscle memory, improves reaction time, and enhances physical toughness. The mental approach emphasizes assertiveness, aggression, and resilience. Physical conditioning, including strength training, impact conditioning, and cardiovascular endurance, is vital for mastering these arts (van Dijk et al., 2014). Hard martial arts are widely used in self-defense, competitive sports, and military or law enforcement training due to their ability to apply overwhelming force quickly and decisively.

### 8.2 Tai Chi

Traditional Chinese martial art with slow, graceful movements, mindful breathing, and mental concentration (Lin and Zhao, 2025). Unlike hard martial arts that rely on explosive power, Tai Chi emphasizes internal energy cultivation (Qi) and relaxation, aiming to harmonize mind, body, and spirit (Mahalakshmi and Shaji, 2024). The practice enhances balance, alignment, flexibility, strength, coordination, and posture, making it accessible to individuals of all ages, especially older adults or those with limited mobility. Research, including studies from the National Center for Complementary and Integrative Health (2024), shows that regular Tai Chi practice improves balance and proprioception, reducing fall risk in elderly populations (Wayne et al., 2025). Tai Chi has also been beneficial for managing symptoms of neurological disorders like Parkinson’s disease, improving motor function, gait stability, and muscle control (Yeung et al., 2018).

## 9 Current research gaps

Despite growing evidence supporting the cognitive and mental health benefits of martial arts for older adults, significant gaps in the literature limit the generalizability of findings and the mechanistic understanding of benefits. A significant limit to the literature is that no standardized protocols are used for interventions across studies (Sullivan et al., 2024a; Sullivan et al., 2024b). Variations in martial arts style, frequency, duration, and intensity in training make it difficult to compare outcomes of studies or determine optimal interventions for various age and health groups. In addition, few studies using randomized clinical trials and long-term follow-up have been conducted to study the sustainability of benefits found for these interventions outside of short-term trials (Moore et al., 2019).

Another area of study that remains sparse is the mechanistic understanding of neurological and biochemical processes associated with the psychological effects of martial arts. While some studies have shown increased levels of brain-derived neurotrophic factor (BDNF), increased neural connectivity, there is insufficient use of biomarkers, electrophysiological tools, and neuroimaging to support differences in mechanisms underlying the benefits of martial arts (Campanella et al., 2024; Lin et al., 2025). Also, gender differences and cultural factors are rarely explored, despite evidence that cultural relevance, social norms, and individual motivation may affect adherence and psychosocial outcomes.

Finally, there is a need for comparative studies of hard versus soft martial arts (Karate, Judo, Taekwondo) versus Tai Chi and Qigong, directed toward the efficacy of enhancing mental health effects, enhanced neural plasticity, and improved emotional regulation. The study of these gaps with interdisciplinary-based rigorous study designs will be crucial in establishing evidence-based guidelines for the efficacy of martial arts and in having these incorporated broadly into public health and aging-related strategies.

## 10 Methodological limitations of current evidence

Despite this increasing literature on the cognitive and psychosocial benefits of martial arts for older adults, several methodological shortcomings limit the strength and transferability of these findings. First, a lot of neuroimaging (and biomarker) studies are based on small sample sizes, which reduces statistical power and increases the risk for false positive findings. The studies that investigate BDNF changes or functional connectivity in Tai Chi practitioners (Sungkarat et al., 2018), commonly use fewer than *n* = 30 participants, which prohibits conclusions about a greater part of the aging population. Moreover, much of the existing research has insufficient control groups in observational studies where individuals self-select into martial arts practice. This introduces the potential for confounding, including level of fitness at baseline, motivations, or cultural familiarity with martial arts (Knight, 2021).

## 11 Future directions

While the positive effects of martial arts on mental health and cognitive function in older adults are well-supported, significant gaps remain in the research. A key limitation is the focus on short- to medium-term interventions (8–16 weeks), offering limited insight into the long-term sustainability of benefits. Future research should prioritize longitudinal studies with extended follow-up periods to assess whether improvements in cognition, mood, and overall mental health persist, increase, or diminish with continued practice or after cessation. Understanding these effects is essential for developing martial arts as a long-term therapeutic strategy for aging populations.

Moreover, longitudinal research could explore martial arts’ potential role in delaying or mitigating neurodegenerative diseases such as Alzheimer’s, Parkinson’s, and dementia. Non-pharmacological interventions like martial arts may offer low-cost options for neuroprotection and symptom management. Investigating the biological mechanisms of martial arts’ cognitive benefits is crucial. Future studies should use neuroimaging and biomarker analyses, such as BDNF, brain connectivity, and neuroplasticity markers, to understand how martial arts impact brain structure and function.

Additionally, future research should examine psychological and social factors, such as stress reduction, social support, and emotional regulation, that may enhance mental health. Standardizing intervention protocols across martial arts styles and populations will improve comparability and reproducibility. Finally, understanding long-term adherence and engagement is essential, with future studies identifying barriers to sustained participation and developing strategies like community-based programs, remote training, or motivational support systems to improve retention. Addressing these gaps will strengthen the scientific foundation of martial arts interventions and maximize their potential for promoting healthy cognitive aging and psychological well-being.

Future endeavors could incorporate advanced technologies such as virtual reality martial art training, AI-driven movement feedback, and neurofeedback devices to track brain activity when engaging in physical activity in real time. This might help improve existing training protocols for effective impacts on performance, as well as possibly improve motivation and personalize the programs designed for different biophysical capabilities in older adults.

## 12 Conclusion

Martial arts provide a comprehensive approach to enhancing mental health and cognitive vitality in older adults. By integrating physical activity, cognitive stimulation, and social engagement, martial arts positively influence neural processes like neuroplasticity and BDNF expression, both crucial for cognitive function with age. Social connections built through martial arts help reduce loneliness and support emotional well-being. Research shows that martial arts training can reduce anxiety and stress, improve executive functions, and enhance quality of life for elderly populations. Both vigorous, dynamic hard martial arts and gentle, mindful soft martial arts like Tai Chi offer unique benefits, addressing physical, cognitive, and psychosocial challenges common in later life. Despite these promising findings, more rigorous studies are needed to clarify the benefits of different martial arts styles, refine training regimens, and assess long-term effects. Comparative and longitudinal research, alongside advanced neuroimaging, will be essential for designing tailored, evidence-based martial arts interventions for older adults. Incorporating martial arts into health promotion programs offers a valuable opportunity to improve mental health, cognitive resilience, and overall well-being, supporting healthier, more fulfilling aging.
